# Peritoneal Mullerian Tumor-Like (Endosalpingiosis-Leiomyomatosis Peritoneal): A Hardly Known Entity

**DOI:** 10.1155/2012/329416

**Published:** 2012-10-08

**Authors:** Rosa Bermejo, Alicia Gómez, Nuria Galiana, Antonio Campos, Rebeca Puente, Ernesto Bas, Carmen Díaz-Caneja

**Affiliations:** ^1^Department of Obstetric and Gynecology, Hospital Marina Baixa, Alicante, 03570 Villajoyosa, Spain; ^2^Department of Pathology, Hospital Marina Baixa, Alicante, 03570 Villajoyosa, Spain

## Abstract

We describe a case of multiple peritoneal nodules with cysts at the free border of the omento and other locations of the peritoneal surfaces. A total abdominal hysterectomy and bilateral salpingooophorectomy were performed for a myomatous uterus and bilateral ovarian cysts. The omentum and several fragments of the abdominal peritoneum were also removed. Microscopic diagnosis was Disseminated Peritoneal Endosalpingiosis-Leiomyomatosis.

## 1. Introduction

Endosalpingiosis is a nonneoplastic lesion of the Mullerian system, similar to but less frequent than the homologous lesions of endometriosis and endocervicosis [1]. They have generally been termed Mullerianosis, including other epithelial proliferations of normal structures of the female genital tract, involving the peritoneum and subperitoneal tissues (uterus, Fallopian tubes, ovaries, bladder, appendix, colon, omentum, pelvic and para-aortic lymph nodes, skin, etc.) [2–4]. The most accepted pathogenesis is metaplastic change of the pluripotential peritoneum cells.

Peritoneal endosalpingiosis, also known as *florid cystic endosalpingiosis *[5], is characterized by the presence of multiple cysts with serous epithelium, frequently ciliated tubal-type epithelium, similar to the epithelium of the Fallopian tubes.

This paper shows multiple peritoneal nodules with solid and cystic areas in the same specimen. It is a rare entity: Disseminated Peritoneal Endosalpingiosis-Leiomyomatosis.

## 2. Case Report

A 44-year-old woman (gravid 3, para 2, miscarriage 1) presented pelvic pain, uterine leiomyomas, and bilateral ovarian tumors. In 1991 an endometriosis cyst was removed. Also myomectomy by hysteroscopy was done in 1999. A total abdominal hysterectomy and bilateral salpingo-oophorectomy were performed. Omento was also removed and multiple biopsies of the abdominal peritoneum were taken. The correct diagnosis was made under microscopic examination. The patient is asymptomatic at the moment, one year after the intervention.

## 3. Pathological Findings

Multiple white mesenteric nodules were found accidentally. Three of the solid ones were submitted as intraoperative biopsy and were informed as benign leiomyomatosis.

**Figure 1:**
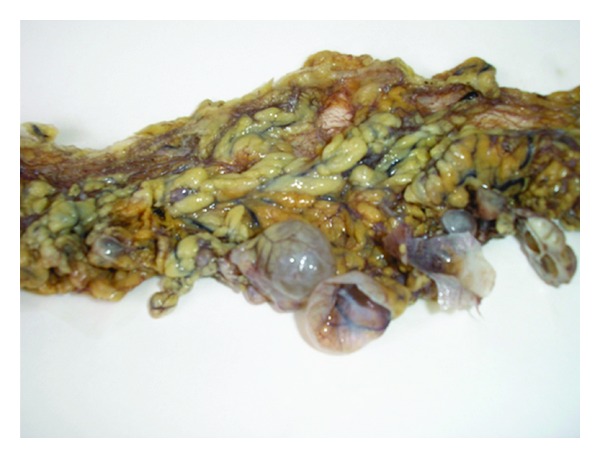
Multiple solid-cystic tumors in the free border of the omentum.

**Figure 2:**
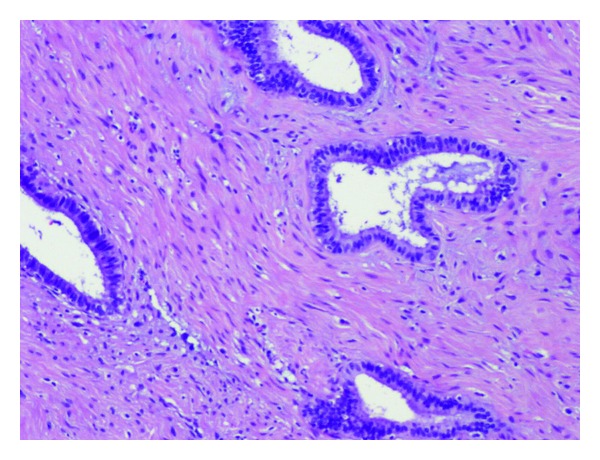
Muscular fibers around tubular structures.

**Figure 3:**
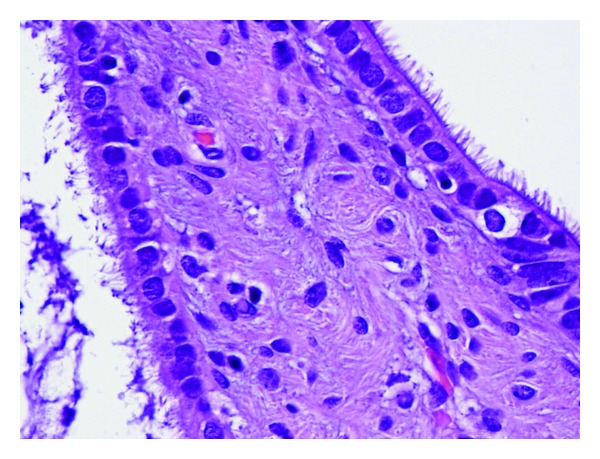
Cystic structures surrounded by ciliated epithelium.

**Figure 4:**
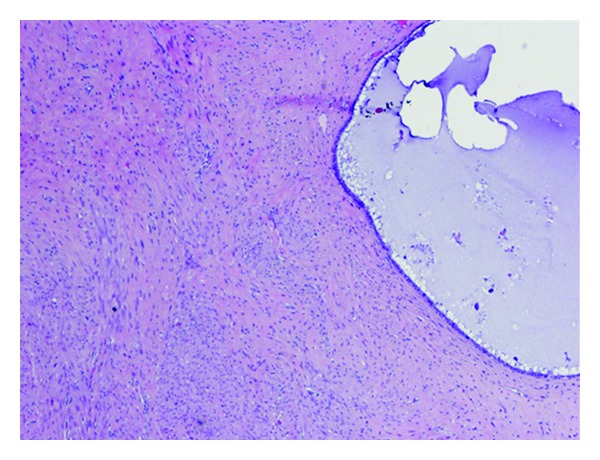
Leiomyomatosis tissue and cysts.

**Figure 5:**
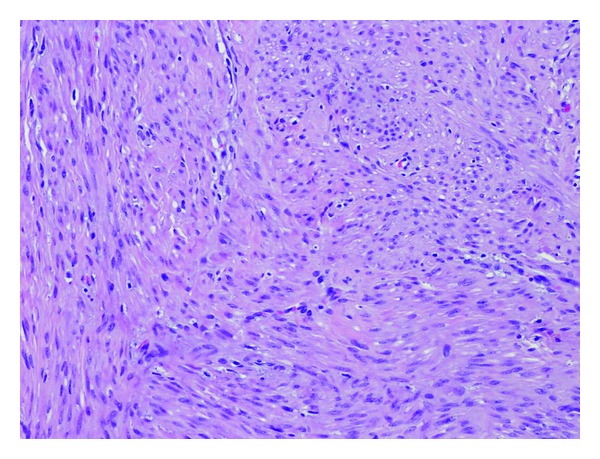
Smooth muscle proliferation.

The surgical specimen consisted of a uterus with multiple leiomyomas and double adnexae with Mature Cystic Teratomas (Dermoid Cyst) in both ovaries. The omento and several biopsies of the abdominal peritoneum were also received. Multiple nodules between 0.2 and 3 cm were found in the free border of the omento. The smaller ones were solid while the others had cystic areas with smooth wall and clear liquid (Figure 1). Microscopic features: cysts were lined by cuboidal or columnar epithelial cell (Figure 2); many of them were ciliated (Figure 3), and surrounded by smooth muscle (Figures 4 and 5). No mitotic figures or atypia were identified. The diagnosis was Peritoneal Endosalpingiosis-Leiomyomatosis, Bilateral Ovarian Mature Cystic Teratomas, and Uterine Multiple Leiomyomas.

## 4. Discussion

Endosalpingiosis is a benign condition of the Mullerian system characterized by the presence of glands lined by ciliated tubal-type epithelium and involves the peritoneum, subperitoneal tissues, omentum, retroperitoneal nodes, and so forth. It was first described by Sampson in 1930 [1]. The most accepted pathogenesis is metaplastic change of coelomic epithelium into tubal-like epithelium. They include lesions with tubal/serous differentiation (endosalpingiosis) and the homologous lesion of endometriosis and endocervicosis.

The morphological appearance of our case resembles a florid cystic endosalpingiosis. Clement and Young described four cases of florid cystic endosalpingiosis presenting as a tumor-like mass [5]. It is characterized by a polypoid mass composed of multiple cysts lined by tubular-type epithelium. They can be differentiated from the usual appearance of endosalpingiosis by the absence of typical cytogenetic endometrial-like stroma and evidence of a cyclical hormonal response (hemorrhage). The authors concluded that endosalpingiosis, although usually is a microscopic finding, can rarely preset as a mass that can be confused with a neoplasm. The cellular stratification and no mitotic activity or atypia contradict the diagnosis of carcinoma.

The symptomatology is not specific. The most common symptoms are pelvic pain, hyper or dysmenorrhea, and infertility but sometimes they have no complaints at all [6, 7]. It seems to be an accidental finding. They are found in association with ovarian tumors [8, 9], endometriosis [10], and myomatous uterus [11]. Our patient presented pelvic pain, uterine leiomyomas, and bilateral ovarian tumors.

In summary, in this paper we describe a case of Omental Endosalpingiosis associated with Disseminated Peritoneal Leiomyomatosis. The importance of our case is to underline the differential diagnosis between these lesions and diffuse peritoneal carcinomatosis or disseminated abdominal malignancy. It represents the crucial diagnostic challenge that can only be made by microscopic exam [12]. Also, sendosalpingiosis can, on rare occasions, present as a macroscopic mass that can be confused with a neoplasm. Our patient is free of disease at the moment, two years after the intervention.
